# Outcomes and predictors of mortality in patients with blunt chest trauma admitted to the intensive care unit: a retrospective study

**DOI:** 10.3389/fmed.2025.1601033

**Published:** 2025-09-10

**Authors:** Ashraf F. Hefny, Ashraf A. Elkamhawy, Sherif A. Fathi, Taoufik Zoubeidi, Fayez Alshamsi

**Affiliations:** ^1^Department of Surgery, College of Medicine and Health Sciences, UAE University, Al Ain, United Arab Emirates; ^2^Department of Surgery, Al Ain Hospital, Al Ain, United Arab Emirates; ^3^Intensive Care Unit, Sheikh Khalifa Medical City, Abu Dhabi, United Arab Emirates; ^4^Department of Surgery, Faculty of Medicine, October 6th University, Cairo, Egypt; ^5^Department of Statistics, United Arab Emirates University, United Arab Emirates; ^6^Department of Internal Medicine, College of Medicine and Health Sciences, UAE University, Al Ain, United Arab Emirates; ^7^Department of Critical Care Medicine, Tawam Hospital, Al Ain, United Arab Emirates

**Keywords:** APACHE II score, blunt chest trauma, lung contusion, mortality, predictor

## Abstract

**Background:**

Blunt chest trauma (BCT) accounts for 25% of trauma-related deaths. we aimed to explore the outcomes and predictors of mortality in patients with BCT admitted to a general intensive care unit (ICU).

**Methods:**

All patients with multiple traumas and BCT who were admitted to the ICU between December 2014 and January 2017 were retrospectively studied. Details on their injuries, demographic characteristics, Glasgow Coma Scale (GCS) score, injury severity score, management, and mortality during ICU admission were retrieved from the hospital trauma registry.

**Results:**

Ninety-two patients were admitted to the ICU. Most cases of BCTs were caused by motor vehicle accidents (75%). Ten patients died (overall mortality: 10.9%). Simple logistic regression analysis identified GCS score, invasive mechanical ventilation, and acute physiology and chronic health evaluation II (APACHE II) score as significant predictors of mortality. Multivariate logistic regression analysis revealed that the APACHE II score was the best predictor of mortality. A one-unit increase in the APACHE II score corresponded to a 17% increase in the odds of death, and an APACHE II score of ≥15 had a sensitivity and specificity of 90 and 81.7%, respectively, in predicting ICU mortality.

**Discussion:**

BCTs were common among patients with polytrauma, and together with concomitant injuries leads to significant ICU resource utilization and worse outcomes.

**Conclusion:**

The APACHE II score, GCS score, and mechanical ventilation were significantly associated with mortality among patients with BCT admitted to the ICU. These factors may be considered for early ICU triage.

## 1 Introduction

Trauma is one of the most common causes of mortality worldwide and is considered as a major health problem with an average 248 million global Disability-Adjusted Life Years in 2021 (1, 2). In the United Arab Emirates (UAE), trauma due to motor vehicle collision (MVC) is one of the leading causes of death (3). In MVCs, chest injuries are common because unrestrained car occupants may be exposed to a greater incidence of blunt chest trauma (BCT) (4, 5). BCT accounts for approximately 25% of trauma-related deaths (6). Rib fractures, lung contusion (LC), pneumothorax, and hemothorax are the most common types of intrathoracic injuries in BCT (4). Moreover, many patients may have concomitant extrathoracic injuries to the brain, abdomen, pelvis, extremities, and cervical regions (6). Patients with BCT require complex multidisciplinary care, which is frequently provided in intensive care units (ICUs) (4, 7) and can lead to significant morbidity and mortality. For example, 60% of patients with multiple fractured ribs in flail chest require invasive mechanical ventilation; among them, 20% develop pneumonia, with mortality reaching as high as 20% (4, 8).

BCT is a diverse entity with different etiologies and distributions across different age groups (9–11). Several factors have been reported to predict outcomes in patients with BCT, including age >65 years, three or more rib fractures, preexisting morbidity, high injury severity score (ISS), need for mechanical ventilation, smoking status, and body mass index (12, 13).

We aimed to study the outcomes and predictors of mortality among patients with BCT admitted to a general ICU in a university-affiliated hospital in the UAE.

## 2 Methods

### 2.1 Patients and data collection

Al Ain Hospital is a university-affiliated community-based hospital with modern trauma and acute care facilities and a mixed medical and surgical ICU. We retrospectively collected the data of all patients who were admitted for BCT from December 2014 to January 2017. The details of patients’ injuries were retrieved from the hospital’s trauma registry, and missing information was manually retrieved from the individual electronic medical records of patients. The data were collected and recorded on a specially designed collection form. The following data were collected: demographic data, vital signs, Glasgow Coma Scale (GCS) score at admission, mechanism of injury, anatomical location and severity of the injury, associated injuries, management, hospital and ICU length of stay, and mortality during ICU admission. The overall injury severity was determined using the ISS, new injury severity score (NISS), and acute physiology and chronic health evaluation II (APACHE II) score. The ISS and NISS were calculated according to the Abbreviated Injury Scale 2008 handbook (14). A comparison between patients with BCT who died and those who survived was performed. This study adhered to the Strengthening the Reporting of Observational Studies in Epidemiology guidelines (15). All patients who were admitted to the hospital or their legal representatives signed the general consent for health services, which includes an agreement for conducting medical or scientific research. This study was approved by the Tawam Human Research Ethics Committee, Abu Dhabi, UAE (T-HREC Ref. No: MF2058-2023-952).

#### 2.2 Statistical analysis

The collected data were entered into a Microsoft Excel spreadsheet (Excel for Mac Version 16.81, Microsoft Corporation, Washington). The data are presented as the mean and standard deviation (SD) or as the frequency and percentage, where appropriate. The Mann–Whitney U test was used to compare two independent groups for continuous or ordinal data, whereas Fisher’s exact test was used to compare two independent groups for binary data. The Chi-square test was used to compare groups for categorical data whenever Fisher’s exact test was not applicable. Statistical significance was considered at *p* ≤ 0.05. The effects of each factor on patient mortality were investigated using simple logistic regression.

Logistic regression analyses were conducted to investigate the association between the various factors and patient mortality. In view of the limited number of events (*n* = 92, number of fatalities = 10), univariable logistic regressions were first performed to examine the association between the response variable (patient’s mortality status) and each predictor individually. The three predictors which demonstrated statistical significance in univariable analyses were subsequently included in a stepwise multivariable logistic regression (forward likelihood ratio) model to identify a parsimonious subset of predictors. This approach is consistent with the recommendation of maintaining a minimum of five events per variable (EPV) to ensure stability of coefficient estimates and mitigate small-sample bias (16). This EPV criterion was applied to minimize the risk of overfitting and to enhance the validity and generalizability of the model results.

Statistical significance was considered at *p* ≤ 0.05. Statistical analyses were performed using the Statistical Package for the Social Sciences (IBM SPSS version 28, Chicago, IL, USA).

## 3 Results

In total, 4,779 patients were included in the Trauma Registry of Al Ain Hospital between December 2014 and January 2017. Among them, 669 (14%) patients had BCT. Of these patients, 92 (13.8%) patients were admitted to the ICU. The mean age (SD) of patients was 30.1 (15.5) years. Twenty-three patients (25%) were Emiratis and represented the most common nationality among the study population, followed by Pakistanis (18, 19.6%), Bangladeshi (10, 10.9%), and Indians (10, 10.9%). The baseline characteristics of the study population are shown in Table 1.

**Table 1:** Baseline characteristics of the study population (*N* = 92).

Key descriptive characteristics	
Demographics	
Males (*n*, %)	81 (88%)
Females (*n*, %)	11 (12%)
Age, years (mean, SD)	30,1 (15.5)
Systolic blood pressure, mmHg (mean, SD)	127 (28)
Pulse, beats/min (mean, SD)	100 (28)
Respiratory rate, breaths/min (mean, SD)	23 (7)
O_2_ saturation, % (mean, SD)	96 (7)
GCS (mean, SD)	12 (4)
Injury details^*^ (*n*, %)	
Lung contusion (*n*, %)	66 (71.7%)
Pneumothorax (*n*, %)	37 (40.2%)
Multiple ribs (*n*, %)	36 (39.1%)
Hemothorax (*n*, %)	18 (19.6%)
First and/or second rib fractures (*n*, %)	12 (13.0%)
Surgical emphysema (*n*, %)	12 (13%)
Single rib fracture (*n*, %)	7 (7.6%)
Sternum fracture (*n*, %)	3 (3.3%)
Heart injury (*n*, %)	3 (3.3%)
Great vessel injury (*n*, %)	3 (3.3%)
Diaphragm injury (*n*, %)	2 (2.2%)
Flail chest (*n*, %)	1 (1.1%)
Injury severity scores (mean, SD)	
ISS (mean, SD)	22 (10)
NISS (mean, SD)	25 (11)
APACHE II (mean, SD)	10 (8)
AIS score chest (mean, SD)	3 (1)
AIS score head (mean, SD)	3.2 (1.3)

* The percentage is more than 100% because some patients have more than one injury.

AIS, Abbreviated Injury Scale; GCS, Glasgow Coma Score; ISS, Injury Severity Score; NISS, New Injury Severity Score.

Work-related injuries were the cause of admission in 19 out of the 90 cases (21.1%). MVC was the most common mechanism of injury (69; 75%), followed by falling from a height of more than 1 m (14; 15.2%). With regard to the type of MVC, car collision was the most common type (49; 53.3%), followed by pedestrian collision (14; 15.2%). Among the motor vehicle occupants who were admitted to the ICU, drivers were the most common (37, 40.7%).

Ten (10.9%) patients were transferred from other hospitals to Al Ain Hospital. Vital signs on presentation and injury severity scores are presented in Table 1.

LC was the most common chest injury in 66 (71.7%) patients (Table 1). The head was the most injured extrathoracic region in 40 (43.5%) patients, followed by the spine in 39 (42.4%) patients. Fifty (54.3%) patients received blood transfusions, and 44 (47.8%) patients were intubated and mechanically ventilated upon ICU admission. The mean (SD) length of stay in the ICU and hospital were 11.2 (14.0) and 18.7 (16.9) days, respectively. Different operative interventions were needed for 48 (52.2%) patients, and a chest tube was inserted for 17 (18.7%) patients.

Mechanical ventilation was needed in numerous cases due to factors such as severe head injury, lung contusion, cardiothoracic injuries and in complications arising during clinical care, including septicaemia, thromboembolic events, and multi-organ dysfunction. The analysis revealed no statistically significant association between chest AIS score and ICU mortality (*p* = 0.62, Mann–Whitney test). However, a noticeable trend was observed indicating higher head AIS scores among non-survivors (*p* = 0.07, Mann–Whitney test), suggesting a potential link between severe head trauma and increased mortality risk.

Ten patients died (overall mortality: 10.9%). Simple logistic regression models of various factors versus mortality in the ICU showed that the GCS score was significantly associated with mortality among ICU patients (Table 2). A one-unit increase in the GCS score was associated with a 23% decrease in the odds of death (*p* = 0.002). Moreover, intubation was significantly associated with mortality, with the odds of death being 12 times greater among intubated patients (*p* = 0.021). In addition, a higher APACHE II score was associated with greater odds of death. A one-unit increase in the APACHE II score corresponded to a 17% increase in the odds of death (Table 2). No statistically significant differences in other categorical variables between surviving and nonsurviving patients were identified (Table 3).

**Table 2:** Simple logistic regression for variables affecting the mortality of patients with BCT in the ICU.

Variable	Coefficient	*p*-value	Odds ratio	95% CI for odds ratio	
				LL	UL
Age	0.019	0.346	1.02	0.98	1.06
Sex = Male	−0.707	0.414	0.49	0.09	2.69
Work-related injury = Yes	0.539	0.469	1.71	0.40	7.38
Systolic blood pressure	−0.006	0.633	0.99	0.97	1.02
Pulse	0.008	0.461	1.01	0.99	1.03
Respiration rate	−0.017	0.777	0.98	0.88	1.10
O_2_ saturation	0.406	0.138	1.50	0.88	2.57
GCS	−0.264	**0.002**	0.77	0.65	0.91
Blood transfusion = Yes	0.259	0.704	1.30	0.34	4.94
ISS	0.057	0.102	1.06	0.99	1.13
NISS	0.047	0.113	1.05	0.99	1.11
Intubation = Yes	2.492	**0.021**	12.09	1.46	99.87
Lung contusion = Yes	−0.598	0.388	0.55	0.14	2.13
Hemothorax = Yes	0.031	0.971	1.03	0.20	5.33
APACHE II score	0.160	**<0.001**	1.17	1.07	1.28
First and/or second rib fractures = Yes	−0.332	0.763	0.72	0.08	6.23

APACHE II, acute physiology and chronic health evaluation II; LL, lower limit; UL, upper limit.Bold values indicates statistical significance; *p* value ≤ 0.05.

**Table 3:** Categorical variables affecting mortality in patients with BCT in the ICU.

Factor	Values	Statistics	Died		*p*-value^b^
			No	Yes	
Sex	Male	Count	73	8	0.342
		Percentage	90.1%	9.9%	
	Female	Count	9	2	
		Percentage	81.8%	18.2%	
Work-related injury	Yes	Count	16	3	0.435
		Percentage	84.2%	15.8%	
	No	Count	64	7	
		Percentage	90.1%	9.9%	
Blood transfusion	Yes	Count	44	6	0.750
		Percentage	88.0%	12.0%	
	No	Count	38	4	
		Percentage	90.5%	9.5%	
Intubation	Yes	Count	35	9	**0.006**
		Percentage	79.5%	20.5%	
	No	Count	47	1	
		Percentage	97.9%	2.1%	
Lung contusion	Yes	Count	60	6	0.460
		Percentage	90.9%	9.1%	
	No	Count	22	4	
		Percentage	84.6%	15.4%	
Hemothorax	Yes	Count	16	2	1.000
		Percentage	88.9%	11.1%	
	No	Count	66	8	
		Percentage	89.2%	10.8%	
Pneumothorax	Yes	Count	35	2	0.169
		Percentage	94.6%	5.4%	
	No	Count	47	8	
		Percentage	85.5%	14.5%	
First and/or second rib fractures	Yes	Count	11	1	1.000
		Percentage	91.7%	8.3%	
	No	Count	71	9	
		Percentage	88.8%	11.3%	
Multiple rib fracture	Yes	Count	34	2	0.305
		Percentage	94.4%	5.6	
	No	Count	48	8	
		Percentage	85.7%	14.3%	

^b^Fisher’s exact test.Bold values indicates statistical significance; *p* value ≤ 0.05.

Stepwise multiple logistic regression (forward likelihood ratio) of the factors that were significantly related to mortality (simple logistic regression results in Table 2) was used to determine the best subset of the predictors of mortality. The results showed that the APACHE II score seemed to be the best predictor of mortality among ICU patients (Table 4).

**Table 4:** Stepwise multiple logistic regression (forward likelihood) of patients with BCT who died in the ICU.

Variable	B	*p*-value	Exp(B)	95% CI for EXP(B)	
				Lower	Upper
APACHE II score on ICU admission	0.143	0.008	1.154	1.039	1.283
Constant	−4.247	0.000	0.014		

Variables in stepwise logistic regression: age, sex, work-related injury, systolic blood pressure, pulse, respiratory rate, O_2_ saturation, GCS, blood transfusion, ISS, NISS, intubation, lung contusion, hemothorax, APACHE II score on ICU admission.

**Figure 1:**
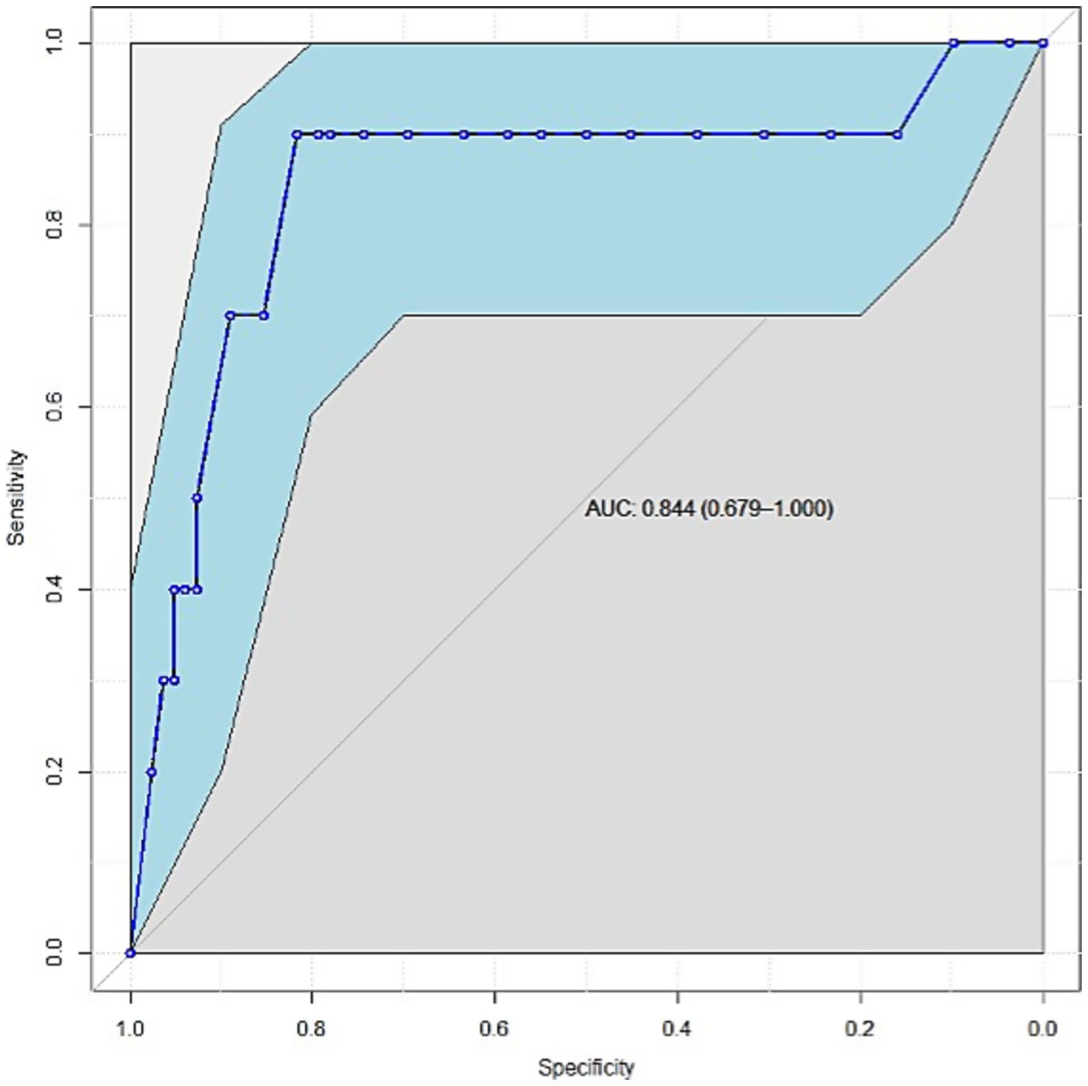
Receiver operating characteristic curve of APACHE II scores for predicting mortality among patients with BCT in the ICU. AUC: area under the curve.

The receiver operating characteristic curve of the APACHE II score as a predictor of mortality had an area under the curve of 0.884 (90% confidence interval: 0.679–1.000), suggesting the excellent ability of the APACHE II score to predict patient survival. The predictive model based on logistic regression correctly categorized 88.4% of ICU patients using the APACHE II score (Figure 1). An estimated probability of death of approximately 0.131 was used as a cutoff point for classifying patients as dead versus surviving, which corresponds to an approximate APACHE II score of 15. This classification scheme had a sensitivity and specificity of 90 and 81.7%, respectively, in predicting the mortality of patients with BCT in the ICU.

## 4 Discussion

The proportion of patients with BCT requiring ICU admission (14%) is comparable to that reported in literature (7.1–36.3%) (17, 18). Many patients with BCT require ICU admission, which can lead to significant healthcare resource utilization. Concomitant cardiothoracic and other serious associated injuries such as severe head injury can explain the need for ICU admission. Furthermore, patients may be admitted to the ICU due to complications arising during clinical care, including sepsis, thromboembolic events, and multi-organ dysfunction. Our findings showed that the GCS score on admission, invasive mechanical ventilation, and APACHE II score were significant predictors of mortality among patients with BCT in the ICU. These findings can help in making clinical decisions when triaging patients and escalating management plans, including early ICU admission (19).

Despite representing 10% of the total population in the UAE, Emiratis were the most commonly admitted patients (25%), followed by Pakistanis (19.6%) (20). A previous study from the Al Ain region reported a similar distribution, wherein Emiratis accounted for 25% (269/1070) of MVC casualties, which was the most common mechanism of injury in the present study (21). One possible explanation for the overrepresentation of Emiratis includes a higher rate of risky driving behavior among young Emiratis, as reported by a recent survey wherein 47.1% of drivers reported not using seatbelts while involved in an MVC in Abu Dhabi (22), drivers were the most commonly admitted motor vehicle occupants (40%). Unrestrained drivers are more likely to suffer chest injuries than other occupants, including front- and rear-seat passengers (21). Several factors may be responsible for the greater severity of chest injury, including injury by rigid structures such as the steering wheel.

Twenty-one percent of patients with serious BCT admitted to the ICU experienced work-related trauma, which may indicate high injury severity in occupational BCT. Health and safety practices at the workplace in the UAE may be responsible for the high incidence and severity of BCT (23). The rapid economic growth in the UAE was associated with an increased number of foreign manual labor workers working on major infrastructure projects. The majority of these workers have a low income, and many may not have proper protective equipment, exposing them to severe injuries. A previous study reported that occupational injuries accounted for approximately 30% of trauma injuries in the UAE, wherein the chest was the second most affected region (13%) (23). Developing a comprehensive occupational safety program is essential for reducing the burden of work-related injuries (24).

Head trauma was the most common extrathoracic injury (44%), which is similar to the findings of other studies (9, 25). This may be explained by the close proximity of the head and thorax regions and the need for invasive mechanical ventilation and ICU admission in many patients with head trauma.

LC was the most common chest injury (*n* = 66, 72%); this finding was similar to that of a large multicenter trauma registry study of 22,613 patients with severe chest injury in Europe (11). A previous study found that LC is associated with worse outcomes in patients with polytrauma, emphasizing the importance of early diagnosis and management (25). Moreover, the clinical manifestations of LC may not be apparent upon presentation until several hours later. Furthermore, a previous study found that plain chest X-ray on admission identified only 50% of LC (26). In addition, other concomitant chest injuries may further lead to delayed diagnosis (25). However, computed tomography scans are more sensitive than X-rays in the early detection of LC (27). The routine use of computed tomography scans in the evaluation of trauma patients helps identify LC almost immediately after injury. According to a previous study, the volume of lung involvement is also correlated with clinical outcomes (28). Thus, approximately half of patients with severe LC require invasive mechanical ventilation (25).

Approximately 48% of the patients were on invasive mechanical ventilation upon ICU admission, and they had higher mortality than patients without invasive mechanical ventilation. There are several pathophysiological mechanisms associated with the worse outcomes of mechanically ventilated patients in our study, wherein 72% had LC. Ventilator-induced lung injury and increased intrathoracic pressure combined with its effect on other organ systems (e.g., the cardiovascular and central nervous systems) may explain the increased mortality in this vulnerable population (29). The mortality in our study was 10.9%, which is comparable to that reported in literature (6–22%) (4, 18).

A study of 127 patients with BCT revealed that the GCS and APACHE II scores were significantly worse in nonsurvivors (30). This finding is similar to the results of the present study, wherein a one-unit increase in the GCS score was associated with a 23% decrease in the odds of death.

Several scoring systems can be used to predict outcomes in patients with polytrauma, and they are broadly categorized based on either anatomy or physiology (31). In one study, the sequential organ failure assessment and APACHE II scores (the latter often readily available for all ICU patients) (32) and the trauma and ISS were comparable (33). However, their evaluation was not exclusive to BCT. Esme et al. compared the revised trauma score, trauma and ISS, lung injury scale score, and chest wall injury scale score in a cohort of 152 patients with BCT admitted to the ICU and reported that the lung injury scale was the most predictive of outcomes (34). Another study proposed the thoracic trauma severity score (35). A very high thoracic trauma severity score has also been associated with the development of acute respiratory distress syndrome (14, 15, 17, 36).

Previous studies investigated the APACHE II score in patients with polytrauma, and the results were comparable to those of other scoring systems (33, 37). Although it has not been specifically studied in the BCT population, we found it to be significantly associated with mortality compared with the ISS and NISS.

An ISS > 15 indicates severe injury and predicts mortality in trauma patients (38). Although the mean (SD) ISS was 22.1 (9.6), it was not a statistically significant predictor of mortality (*p* = 0.082). The ISS is valuable for evaluating the injury severity in different anatomical regions of the body. However, it does not fully incorporate the physiological aspects of trauma, such as shock or altered mental status, chronic health conditions, and age effect. Patients who need admission to ICU may have similar ISS, although their physiological status may greatly differ (39). This finding may highlight the importance of physiological parameters in predicting mortality over anatomical parameters.

Our study has several limitations. First, the study’s retrospective nature is prone to selection bias and incomplete data. The effect of these was ameliorated by including all patients in the registry and ensuring accurate and consistent data collection methods, including manual retrieval of missing information from electronic medical records. Second, our sample size (*n* = 92) was small, leading to an overall low event rate (deaths, *n* = 10). This can affect the robustness of our findings on the performance of the APACHE II score as a predictor of mortality. We used statistical methods to mitigate this. However, few studies have used the APACHE II score for this purpose in this context. On the other hand, the study has several strengths. Our trauma registry complied with the National Trauma Data Bank of the American College of Surgeons Committee on Trauma. We included all consecutive patients with BCT within the study period. The demographic data of our population, including the mechanisms of injury and outcomes, are comparable to those reported in literature. We used a robust statistical analysis plan to identify the predictors of patient-important outcomes, including mortality. Nevertheless, a prospective multicenter study is warranted for validation of our results.

## 5 Conclusion

BCT with severe associated injury will increase the morbidity and mortality, and ventilation support prior to or upon ICU admission. The outcome predictors included invasive mechanical ventilation, GCS score upon admission, and APACHE II score. An APACHE II score of ≥15 had a sensitivity and specificity of 90 and 81.7%, respectively, in predicting mortality. As such, an APACHE II score of ≥ 15 should trigger early ICU admission. The APACHE II score, which is often readily available for critically ill patients, was the best predictor of mortality and may play a promising role in triaging and predicting outcomes in this population.

## Data Availability

The original contributions presented in the study are included in the article/supplementary material, further inquiries can be directed to the corresponding author.
